# Lightweight Structural Biomaterials with Excellent Mechanical Performance: A Review

**DOI:** 10.3390/biomimetics8020153

**Published:** 2023-04-12

**Authors:** Zhiyan Zhang, Zhengzhi Mu, Yufei Wang, Wenda Song, Hexuan Yu, Shuang Zhang, Yujiao Li, Shichao Niu, Zhiwu Han, Luquan Ren

**Affiliations:** 1 Key Laboratory of Bionic Engineering, Ministry of Education, Jilin University, Changchun 130022, China; 2 Weihai Institute for Bionics, Jilin University, Weihai 264207, China

**Keywords:** lightweight structure, biomaterials, bioinspired design, mechanical performance

## Abstract

The rational design of desirable lightweight structural materials usually needs to meet the strict requirements of mechanical properties. Seeking optimal integration strategies for lightweight structures and high mechanical performance is always of great research significance in the rapidly developing composites field, which also draws significant attention from materials scientists and engineers. However, the intrinsic incompatibility of low mass and high strength is still an open challenge for achieving satisfied engineering composites. Fortunately, creatures in nature tend to possess excellent lightweight properties and mechanical performance to improve their survival ability. Thus, by ingenious structure configuration, lightweight structural biomaterials with simple components can achieve high mechanical performance. This review comprehensively summarizes recent advances in three typical structures in natural biomaterials: cellular structures, fibrous structures, and sandwich structures. For each structure, typical organisms are selected for comparison, and their compositions, structures, and properties are discussed in detail, respectively. In addition, bioinspired design approaches of each structure are briefly introduced. At last, the outlook on the design and fabrication of bioinspired composites is also presented to guide the development of advanced composites in future practical engineering applications.

## 1. Introduction

With the rapid development in the field of modern engineering materials, strict material design requirements are proposed to take both lightweight properties and high mechanical performance into account. However, it is unrealistic to perfectly integrate these mutually exclusive properties for most conventional artificial materials [1,2,3,4,5]. For instance, the lightweight design of materials inevitably leads to lower density, thus weakening their mechanical performance. This dilemma has become a thorny scientific problem limiting the industrial application of many lightweight materials [6]. To resolve this conflict, materials scientists have looked to natural biomaterials with excellent mechanical performance to design new structural materials to meet engineering requirements [5,7,8,9,10].

Typical biological prototypes that live in harsh natural environments have evolved for billions of years to form excellent biomaterials with elaborate structures. Interestingly, due to their carbon-based main components, biomaterials have the natural quality of being lightweight. Moreover, naturally optimized structure configurations of biomaterials build the foundation for the improvement of their mechanical properties, which allow biomaterials to achieve the near-perfect integration of lightweight, high strength, and excellent toughness [7,11,12]. For example, birds have evolved a variety of cellular structures to ensure flight stability [13,14,15]; shells have evolved excellent interfaces for high mechanical properties with lower masses [16,17]; antlers have multilevel porous structures inside the cancellous bone and dense cortical bone to resist local buckling with minimal mass [18,19]. The giant bird of paradise (*Strelitzia*) plant stem features a hollow structure inside, with rectangular cells in the longitudinal section and radially arranged cell walls in the transverse section. The structure is designed to resist flexural stress without buckling [1].

**Figure 1:**
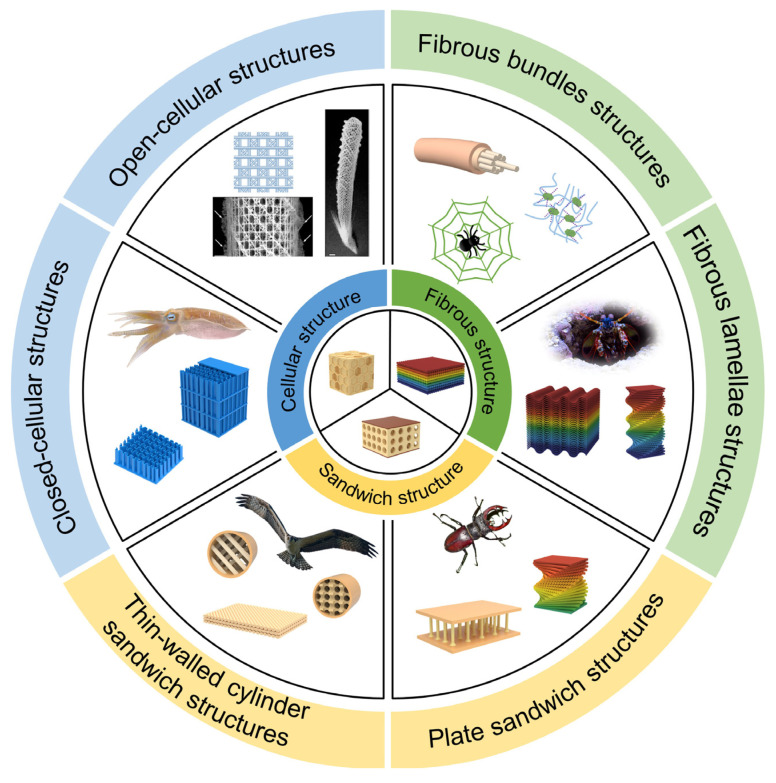
Three typical structures of lightweight structural biomaterials and corresponding biological prototypes. These structures are mainly subdivided into open-cellular structures and closed-cellular structures; fibrous bundles structures and fibrous lamellae structures; plate sandwich structures and thin-walled cylinder sandwich structures. Reproduced with permission from J. Aizenberg et al. [23], American Association for the Advancement of Science. Whenever relevant, images were reproduced with permission from the website.

Hence, lightweight structural biomaterials offer the possibility and inspiration of manufacturing composites with high mechanical performance. However, most existing composites are limited to mimicking the basic morphologies and structures of natural creatures. There is still a long path to learning the deep intrinsic mechanisms of typical biological prototypes [4,20,21,22]. According to the structural forms of selected biomaterials with lightweight and high mechanical performance, this review classifies them into three categories: cellular structure materials, fibrous structure materials, and sandwich structure materials (Figure 1). Moreover, the bioinspired strategies and fabrication processes of the above-mentioned materials are briefly discussed. Finally, the conclusion and outlook on the future development trend of bioinspired composites with lightweight and high mechanical performance are provided.

## 2. Cellular Structure Materials

Cellular structure materials are featured with low density due to their unique porous structures; thus, they are naturally lightweight in mass. Generally, they can be classified into open-cellular and closed-cellular structures according to the structural forms of internal pores and their interconnection. Further they are further classified into periodic and stochastic structures according to the randomness of internal pores [6]. For instance, glass and luffa sponges possess visible open pore structures, while internal microfibers have different structure forms [23,24]. Pomelo peel and cuttlebone have typical multiscale closed-cellular foam structures and honeycomb structures, respectively [25,26]. Foam structures have stochastic internal pores, while honeycomb structures have periodic properties [6].

### 2.1. Open-Cellular Structures

**Figure 2:**
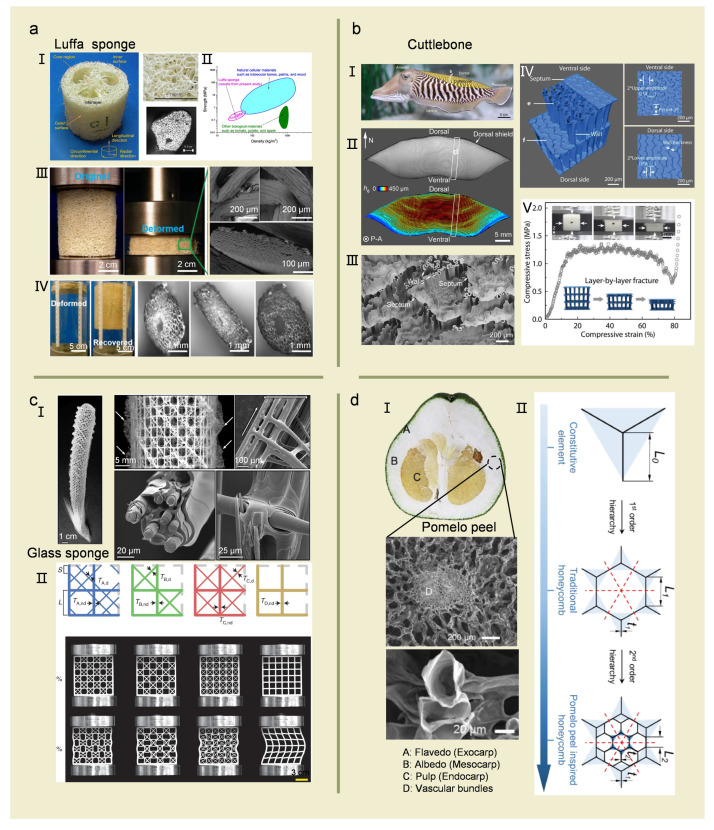
Cellular structures and their typical features in representative biomaterials. (**a**) The structures and performances of luffa sponge. (**I**) Macrostructures and microstructures of luffa fibers. (**II**) Comparison between luffa sponge and other biomaterials. Reproduced with permission from J.H. Shen et al. [24], Elsevier. (**III**) A luffa sponge permanently deformed after axial compression. (**IV**) The rapid water-driven shape recovery of luffa sponge. Reproduced with permission from H.C. Quan et al. [29], Springer Nature. (**b**) The chambered wall-septa structure and performances of cuttlebone. (**I**) Cuttlefish with the cuttlebone. (**II**) Cross-section of the cuttlebone (top) and the corresponding map of chamber height (bottom). (**III**) The walls and septa of the layered chambers SEM image. Reproduced with permission from E. Lee et al. [41], Elsevier. (**IV**) 3D structure of a two-layer model reconstructed from micro-CT images, the cross-sectional views of the walls at the ventral (top) and dorsal (bottom) sides. (**V**) Compressive stress-strain curve of cuttlebone. Reproduced with permission from A. Mao et al. [40], John Wiley and Sons (**c**) Structure analysis and bionic design of the mineralized skeletal system of *Euplectella aspergillium*. (**I**) Macrostructures and microstructures of *Euplectella aspergillium*. Reproduced with permission from J. Aizenberg et al. [23], American Association for the Advancement of Science. (**II**) Bionic design of sponge-inspired lattice geometry. Reproduced with permission from M.C. Fernandes et al. [35], Springer Nature. (**d**) The structures and bionic design of the pomelo peel. (**I**) Macrostructures and microstructures of pomelo peel. (**II**) Hierarchical honeycomb inspired by pomelo peel. Reproduced with permission from W. Zhang et al. [26], Elsevier.

A sponge features a fibrous skeleton composed of calcium carbonate, silica, and protein fibers (spongin) [3]. Luffa sponge, as a kind of cellular material, is from the *Luffa Cylindrica* plant, and its skeleton is mainly composed of protein fibers [27]. Moreover, luffa fibers have a multiscale porous structure (Figure 2a). The structural hierarchy of the luffa sponge includes columns (50 mm), fibers (1 mm), and cellular walls (0.01 mm). Shen et al. [24] investigated the strength and energy absorption properties of luffa sponges. The compressive strength of luffa sponges was about 0.27 MPa, and the corresponding stress-strain curve showed nearly constant plateau stress in the long strain range. It indicated that luffa sponges could be ideal candidates for energy absorption applications. The deformation of the luffa sponge is determined by the coupling of axial compression/tension and luffa fiber bending. Meanwhile, luffa sponges possess excellent self-healing ability due to their multiple graded structures. Shen et al. [28] demonstrated reversible shape changes of the luffa sponge (*Luffa aegyptiaca*) in response to humidity. The graded porous structures of the luffa sponge allow the sponge to deform and crush. However, upon hydration, the luffa will regain its original shape (Figure 2a), which allows it to float and carry the seeds away from their origin. Since plant tissues are highly hydrophobic, the deformed sponge expands its cellular structure by absorbing water through the straightening of the struts and can rapidly recover its original shape (within 10 min) [29].

The glass sponge of the siliceous Venus flower basket (*Euplectella aspergillium)* skeletal system consists of amorphous hydrated silica arranged in a highly regular and stratified cylindrical lattice with excellent flexibility and resistance to damage [3,30,31]. Due to the regular arrays of longitudinal, radial, and helical fibers, these fibrous skeletons are further organized into a highly regular square lattice. These spicules are reinforced by two sets of intersecting diagonal struts forming a checkerboard-like cell pattern of alternating open and closed cells (Figure 2c) [23]. The elastic modulus, fracture stress, and bending toughness of the glass sponge can reach up to 40.82 ± 9.65 GPa, 3727.12 ± 660.77 MPa, and 69.45 ± 11.71 MPa, respectively [32]. The excellent mechanical performance of the glass sponge can be attributed to external toughening in the form of crack deflection and crack twisting when the needle tip breaks [33,34]. This remarkable hierarchical structure and mechanical rigidity over scales have attracted considerable attention from the engineering and materials science communities. Fernandes et al. [35] combined finite element simulations and mechanical tests on 3D-printed specimens with different lattice geometries. The results demonstrated that the diagonal reinforcement strategy of the sponges achieves the highest flexural resistance for a given amount of materials. Evolutionary optimization algorithms were applied to confirm that the sponge-inspired lattice geometry is close to the optimal material distribution for the considered design space. The sponge skeletal systems can inspire the achievement of highly optimized square lattice geometries to avoid global structural buckling, thus improving the mechanical performance of materials applied in modern infrastructures (Figure 2c).

### 2.2. Closed-Cellular Structures

Cuttlebone is a biomineralized shell from the marine mollusk cuttlefish. It has periodically distributed close-cellular structures with excellent mechanical properties [36]. Although the main component of cuttlebone is the friable mineral aragonite, the complex porous structure is highly resistant to damage and can withstand a water pressure of ~20 atm [37]. It can work as a rigid floating box for cuttlefish to resist hydrostatic pressure in the deep-sea environment [36]. Cuttlebone consists of a mixture of aragonite, *β*-chitin, and other protein composites [38,39]. Cuttlebone includes two major structural elements: the dorsal shield and the chamber. The structure of cuttlebone resembles a lamellar compartment separated by asymmetric, twisted “S” shaped walls, exhibiting higher strength and stronger energy absorption properties than octahedral lattice trusses, conventional polymers, and metallic foams (Figure 2b) [25]. The total density of cuttlebone is about 0.2 g/cm^3^ with a porosity of 90%. It consists of lamellar compartments separated by continuously curved walls uniformly distributed in each layer to form channels for internal liquid flow. These lamellar partitions are 300–500 μm height, 8–10 μm thickness, and 80–180 μm in spacing [40]. Different structure units of the cuttlebone show significant structural, chemical, and mechanical variations. In particular, the dorsal shield consists of two hard lamellar and prismatic mineral tissues, which contain more ductile and flexible lamellar structures [39]. A similar organization is found in the chambers, which are separated by a septum and supported by flexural plates. Similar to the dorsal shield, the septum consists of two layers, a laminar and a prismatic organization, which differ significantly in their mechanical properties. The prismatic layer is three times stiffer and up to ten times stiffer when compared to the lamellar tissue. The combination of high stiffness, hardness, flexibility, and toughness may reduce the risk of catastrophic damage, reflecting the role of the organism in the growth of cuttlebone. Mechanically weaker units may act as a sacrificial structure, ensuring the progressive failure of individual chambers in the event of overloading and guaranteeing structural integrity [25,41]. Yang et al. [25] revealed the mechanism by which cuttlebone achieves high energy absorption and damage tolerance through its asymmetric wall fracture, extensive densification, and chamber-by-chamber disruption. Meanwhile, the relationship between the macroscopic response of cuttlebone and its microstructure is established analytically to reveal the biological mechanism of lightweight, high stiffness, and high energy absorption of cuttlebone. Yang et al. and Mao et al. [25,40] proposed several important strategies for the design of cuttlebone in ceramic porous solid materials and lattice metamaterials. The corrugated wave walls have near-straight wall stiffness and can control the maximum stress at specific locations, providing a design pathway for crack path extension. Asymmetric structural features such as the corrugated gradient of the cuttlebone introduce asymmetric fracture and directional damage propagation mechanisms. This approach can be used to improve the mechanical properties of ceramic dotted materials.

Pomelo is a typical biological prototype with stochastically distributed multiscale foam structures (Figure 2d). Mature pomelos (*Citrus max*), weighing up to 6 kg, grow on trees up to 15 m in height and have considerable dynamic energy absorption when they fall to the ground [26]. Thielen et al. [42] demonstrated that the pomelo peel is a gradient structure consisting of an outer epidermis followed by a thin layer of dense cells (flavedo) and a thicker layer of less dense cells (albedo) that protects the fruit from impact damage. Experimental results indicated that up to 90% of the impact energy is dissipated during the free fall of the pomelo. Fischer et al. [43] showed the excellent impact resistance of pomelo peel by free fall testing. Zhang et al. [26] demonstrated that there is a hierarchy of pore sizes and shapes across the thickness of the peel. The narrowest pores are present near the outer surface of the peel. The pores become larger and more open along the distance of the outer surface of the peel. In the transition zone from the pomelo peel to the inner medulla, the pores become smaller but flat and extended in shape. In addition, the pomelo peel has many fibrovascular bundles that serve as struts of the peel. Each bundle includes several interconnected biological cells. The cells are fluid-filled and have multiple layers of walls. Zhang et al. [44] reported that the hydration content of grapefruit has a great influence on the compression properties of the peel. Compared to fresh pomelo peel, dried peel samples exhibited higher compressive modulus and energy loss in the 6, 8, and 10% maximum strain hysteresis tests. By combining X-ray tomography imaging techniques with digital volume correlation, the exploration of the internal mechanisms of structure-function relationships reveals that the bundle of blood vessels plays a critical role in the resistance properties of pomelo peel. This structure could provide significant inspiration for the design of hierarchical porous foam materials [45].

## 3. Fibrous Structure Materials

Most biomaterials can be regarded as composites to a certain extent, including at least two phases with different mechanical properties. Most commonly, samples such as fiber-reinforced composites are rigid reinforcements embedded in a matrix, usually with a large aspect ratio and preferentially aligned within the matrix along a specific orientation [46,47,48,49]. There are various examples of fibrous structures in nature, which can be generally classified into fibrous bundles (spider silk) [50,51,52] and fibrous lamellae. Further, fibrous lamellae can be divided into general laminar structures (e.g., wood [49,53] and bone [20]) and Bouligand structures (e.g., fish scales [54,55] and arthropod exoskeletons [56,57,58,59]).

### 3.1. Fibrous Bundles Structures

Spider silk is a typical natural lightweight and tough filamentous material, which consists of only simple proteins [60,61,62]. Spider silk has remarkable mechanical characteristics, such as an initial modulus of up to 10 GPa, elongation at break exceeding 50–60%, a tensile strength of 1–2 GPa, and a toughness exceeding that of all other natural or synthetic fibers [63,64]. Spider silk density is extremely small, but its ultimate strength is comparable to that of engineering materials such as steel. Moreover, the specific strength of spider silk can be up to 10 times higher than that of steel [33,60,65,66]. The investigation on the toughening mechanism of spider silk indicated that the soft and hard arrangement in spider silk is the key factor for its high tensile strength capability.

**Figure 3:**
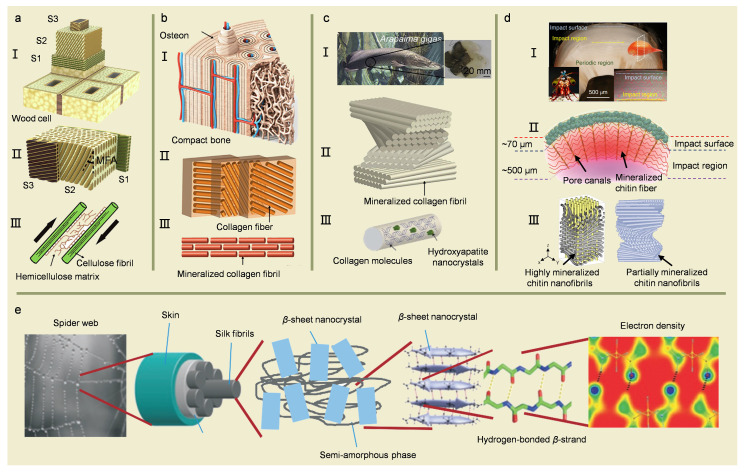
Fibrous structures and their typical features in representative biomaterials. (**a**) Structural features of a wood cell wall. (**I**) Schematic of a tracheid in wood xylem. (**II**) Fibrous lamellae structure of wood cell wall. (**III**) Cellulose fibril and hemicellulose matrix of a wood cell wall. Reproduced with permission from Z.Q. Liu et al. [49], Elsevier, (**b**) Hierarchical architecture of bone. (**I**) Schematic of bone. (**II**) Fibrous lamellae structure of compact bone. (**III**) Mineralized collagen fibrils. Reproduced with permission from Z.Q. Liu et al. [49], Elsevier. (**c**) The hierarchical structure of *Arapaima gigas* scales. (**I**) Overlapping *Arapaima* scales. Reproduced with permission from E.A. Zimmermann et al. [54], Springer Nature. (**II**) Bouligand-type arrangement fibrous lamellae structure. Reproduced with permission from Z.Q. Liu et al. [49], Elsevier. (**III**) Mineralized collagen fibrils. Reproduced with permission from E.A. Zimmermann et al. [54], Springer Nature. (**d**) The hierarchical structure of the mantis shrimp. (**I**) Impact surface of the dactyl club of the mantis shrimp. (**II**) Nanoarchitectural design features of particles within the impact surface of the dactyl club. Reproduced with permission from W. Huang et al. [57], Springer Nature. (**III**). Mineralized chitin nanofibrils arranged in a herringbone pattern of the impact region, and partially mineralized chitin nanofibrils assembled in a helicoidal structure of the periodic region. Reproduced with permission from S.J. Ling et al. [78], Springer Nature. (**e**) Schematic of the hierarchical spider silk structure that ranges from the nanoscale to the macroscale. Reproduced with permission from S. Keten et al. [52], Springer Nature.

From the structural point of view, spider silk exhibits a hierarchical structure as well. The nanoscale *β*-sheet crystals are composed of highly conserved poly-(Gly-Ala) and poly-Ala domains. Then, these *β*-sheet nanocrystals are connected with hydrogen bond assemblies and enmeshed in a semi-amorphous protein matrix made up of less orderly *β*-structures, helices, and *β*-turns (Figure 3e) [52]. The excellent mechanical performances of spider silk depend mainly on the synergistic effect of hydrogen bonding and protein secondary structures. The crystalline regions increase the strength, and the amorphous regions ensure high toughness and ductility. From the perspective of toughening mechanism, low stress corresponds to the unfolding and straightening of protein chains [33,66,67,68,69,70]. After reaching the yield point, as the stress increases, the proteins in the amorphous region of the fiber are gradually expanded and aligned during stretching while the nodes of the protein skeleton and molecular network are stretched to support the load. At this point, the load is transferred to the *β*-sheet nanocrystals in the silk fibers, which causes the expansion of the *β*-sheet nanocrystals and leads to strain hardening of the spider silk. *β*-sheet nanocrystals can tolerate ductile damage rather than brittle damage. High strength and rigidity of spider silk are ensured throughout the spider silk destruction process due to the high cohesive energy density of hydrogen bonds and the physical properties of *β*-sheet nanocrystals, i.e., the synergistic effect of hydrogen bonds and size effect. During the stretching process, the protein chains in the amorphous region are gradually released by the hydrogen bond breakage. They are able to absorb extensive energy during the deformation process, which endows spider silk with high ductility [50,51,52].

### 3.2. Fibrous Lamellae Structures

In the case of fibrous lamellae of wood, bone, fish scale, and arthropod exoskeleton, the reinforcing fibers are mainly in the form of cellulose protofibrils in wood, mineralized collagen fibers in bone and fish scale, and chitin fibers in arthropod exoskeleton. The matrix phase is hemicellulose in wood cell walls (Figure 3a), non-collagenous proteins in bone (Figure 3b) and fish scales (Figure 3c), and proteins in arthropod exoskeletons (Figure 3d). They are mainly present at the interface between the components [49]. In contrast to artificial materials, the interfacial matrix in biomaterials plays a substantial role in the deformation process by redirecting the reinforcing materials [11,20,71].

Bones are characterized by a coaxially layered interlocking structure consisting of 30–43% minerals, 32–44% proteins, and 15–25 vol.% water. Among the proteins, type I collagen accounts for 90%, and others are non-collagenous [2]. There are two typical categories of bones: compact bone and spongy bone. Compact bone exhibits anisotropic behaviors under compressive and tensile loading, which results from the complexity of the layered arrangement and orientation of the bone structural elements. Compact bone exhibits elastic damage behavior under compressive loading [20]. The compact bone has a complex hierarchical structure (Figure 3b), collagen particles, and hydroxyapatite (HA) nanocrystals [20,72,73]. The collagen particles are tropocollagen, approximately 300 nm in length and 1.5 nm in diameter. HA nanocrystals are plate-like: 50 nm × 25 nm in size and 1.5–4 nm in thickness [20,72,73]. These structures are periodically misaligned into collagen fibers parallel to the *c*-axis. Single collagen molecules interact through hydrogen bonding. Collagen fibers are rigid due to the alignment of collagen molecules and the reinforcement of HA nanocrystals. Collagen fibers consisting of bundles of collagen fibers are arranged into crossed layers and lamellae [20,74]. The lamellae are subsequently wrapped concentrically around the Haversian canals to form the osteons. The interface between the bones is called the cement line, which is composed of high levels of minerals and low levels of collagen. The osteons and interfaces further form the compact skeleton [2]. The compact bones exhibit excellent mechanical properties with a modulus of elasticity of 15–20 GPa and tensile strength of 100–160 MPa [2,75]. The intrinsic toughening mechanisms of this structure against fracture are molecular uncoiling and intermolecular sliding of a molecule of collagen, fibrous sliding of collagen bonds, and microcracking of the mineral matrix. The extrinsic mechanisms are hindering crack growth, which includes collagen fiber bridging, ligament bridging for fracture, and crack deflection distortion [3,20].

Chitin is the second most abundant biomass material in nature. It has very similar structural characteristics to cellulose in woody fiber materials in terms of chemical nature and microstructure, which are both polysaccharide biomass materials. Chitin is usually found as a component of the exoskeleton of arthropods or crustaceans, such as shrimps and crabs. It is also involved in the formation of the cell walls of fungi. Generally, it is combined with proteins and calcium to form a hard-composite structure, which acts as an external armor for the organism [56,57,58,59]. In the case of the dactyl club of mantis shrimp, it takes advantage of different areas with various properties and functions to produce strong impact resistance [5,76]. The mineralization level shows a clear gradient, and a high mineralization level exhibits a large number of mineral nanoparticles without fibers near the impact surface. As a result, the hardness and modulus increase towards the surface, reaching a maximum at the impact surface and withstanding large impact forces of up to 1500 N [58,77]. The impact zone, characterized by highly mineralized chitin fibers with a herringbone pattern, is located under the impact surface. It is primarily in charge of simultaneously redistributing stresses and strains to minimize major damage in specific locations. The periodic zone below the highly mineralized impact zone consists of chitin fibers with a unique spiral organization called the “Bouligand” structure, which allows cracks to be transmitted in a helical manner rather than by catastrophic propagation (Figure 3d) [57,78]. Preventing cracking is the main toughening mechanism of this “Bouligand” structure. When subjected to external forces, cracks cannot propagate along a linear path, increasing the material toughness. When subjected to stress, the fibers break, and the chitin fibers can absorb the strain so that the fracture region is not subjected to physical separation with the dispersion of fragments. In general, the dactyl club of mantis shrimp exhibits a gradient mechanical performance through the mineralization degree and fiber arrangement. In addition, the organisms ingeniously combine these different zones to achieve significant impact resistance [5,56,58,59,76].

## 4. Sandwich Structure Materials

Sandwich structure is another excellent lightweight structure that evolved in nature. It possesses both an inner layer with a multi-cellular structure and an outer layer with a fibrous structure, ensuring that the material has a low mass and high mechanical performance [1]. The sandwich structures can be divided into plate sandwich structures and thin-walled cylindrical sandwich structures, which have different mechanical properties.

### 4.1. Plate Sandwich Structures

The beetles, a general term for coleopterans, are widely investigated for their lightweight, damage resistance, and high impact resistance [79,80]. The beetles are featured with a typical plate sandwich structure, which is similar to other arthropods with a fibrous Bouligand structure for the exoskeleton [80]. The interior of beetles has a cavity structure that can be reduced into a smaller volume when subjected to moderate compression, protecting the organs from damage [81].

**Figure 4:**
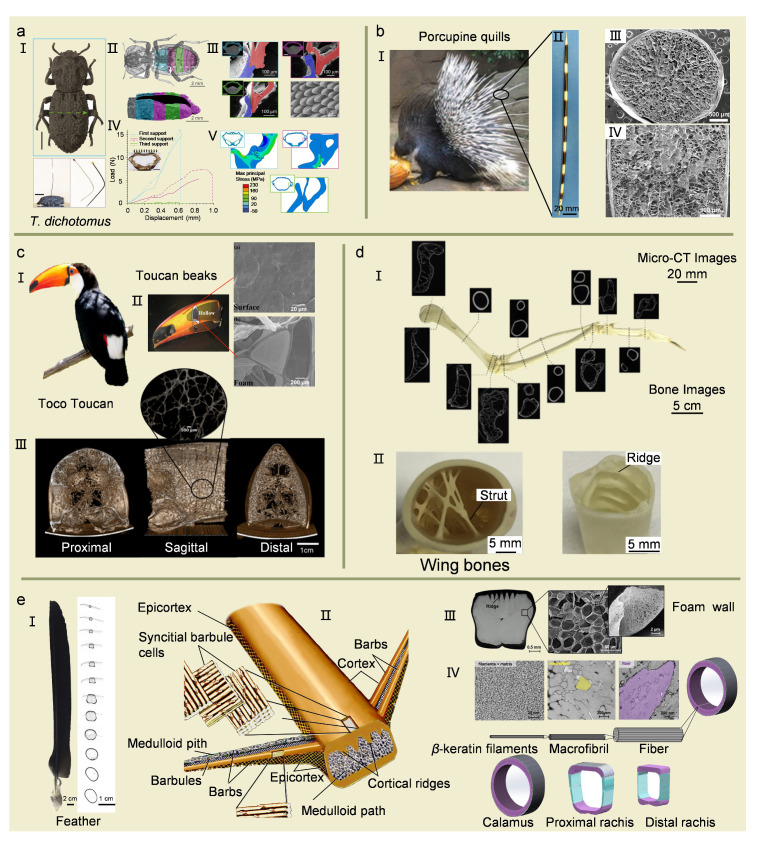
Typical biomaterials of sandwich structures. (**a**) Mechanical and structural characterization of *Phloeodes diabolicus*. (**I**) Image of DIB. (**II**) Plan view and longitudinal CT scans. (**III**) Distinct lateral interfacial architectures between the elytra and ventral cuticle: interdigitated, latching, and free-standing motifs. (**IV**) Compression tests of the entire exoskeleton of the DIB. (**V**) Compression simulations of the entire exoskeleton of the DIB. Reproduced with permission from J. Rivera et al. [82], Springer Nature. (**b**) Porcupine quill exhibiting the dense outer cortex surrounding a uniform, closed-cell foam. (**I**) Photographs of porcupines. Reproduced with permission from M.A. Meyers et al. [1], American Association for the Advancement of Science. (**II**) Photographs of quills. Reproduced with permission from W. Yang et al. [83], Elsevier. (**III**) Entire morphology in a transverse orientation. (**IV**) Image of the longitudinal cross-section. Reproduced with permission from J. McKittrick et al. [91], Springer Nature. (**c**) Toucan beak showing the porous interior with a central void region. (**I**) Schematic diagram of a Toco Toucan. Reproduced with permission from S.G. Bodde et al. [92], Elsevier. (**II**) Rhamphotheca on the exterior surface and the trabecular closed-cell form in the interior of Toucan beaks. (**III**) Three-dimensional foam structure of Toucan. Reproduced with permission from Y. Seki et al. [84], Elsevier. (**d**) The skeletal system of a bird wing. (**I**) Micro-computerized tomography scans of the wing bones of Turkey Vulture (*Cathartes aura*). (**II**) Ridges in the bone. Reproduced with permission from T.N. Sullivan et al. [15], Elsevier. (**e**) Macrostructures and microstructures of flight feathers. (**I**) The shape change along the length of the feather shaft from circular to rectangular. Reproduced with permission from T.N. Sullivan et al. [15], Elsevier. (**II**) Microstructural fiber model of the feather shaft and barbs. Reproduced with permission from T. Lingham-Soliar et al. [93], Springer Nature. (**III**) Ridges and medullary foam of the feather. Reproduced with permission from T.N. Sullivan et al. [15], Elsevier. (**IV**) The hierarchical structure of the feather shaft cortex. Reproduced with permission from T.N. Sullivan et al. [94], Elsevier.

Jesus Rivera et al. [82] discovered a diabolical ironclad beetle (DIB), *Phloeodes diabolicus,* with remarkable resistance to compression. It exhibited considerably stronger stiffness than other beetles of the same species when subjected to longitudinal compression. There was a sudden increase in the stiffness of its exoskeleton after a certain level of compression. Other beetle exoskeletons were only half to one-fifth as stiff as the diabolical ironclad beetle. Moreover, they did not exhibit a sudden increase through the compression process. The exoskeleton of most beetles breaks with a maximum load of 68 N, while the value of the diabolical ironclad beetle can reach up to 149 N (~39,000 times its body weight). The excellent compression resistance of its sheath wings is due to two mechanisms. On the one hand, interdigitated, latching, and free-standing supports connect the sheath wings to the abdominal cortical layer. On the other hand, a medial suture permanently fuses the two sheath wings together. Rod-like protrusions on its surface increase the contact friction and prevent the interlocking edges from slipping out. The combination of these features allows the sheath wings to deform more gently, thereby dissipating energy more evenly and preventing the exoskeleton from breaking suddenly (Figure 4a).

### 4.2. Thin-Walled Cylinder Sandwich Structures

Natural thin-walled cylindrical structures have a thin solid outer shell as well as a lightweight porous structure inside, such as bird wing bones [15], flight feathers [13], porcupine spines [83], beaks [84], and hedgehog spines [85]. Flexure and buckling must be effectively controlled to avoid instability and excessive deformation for long-lasting usage of natural biomaterials. Biomaterials must withstand high loads caused by the external environment and have a defined flexure to effectively avoid catastrophic damage.

Porcupines can defend themselves against predators with dorsal spines that are lightweight and stiff enough to withstand significant compression and bending loads [1,3,11,83]. Porcupine quills have a thin layer of cortex filled with a closed foam that is composed entirely of keratin (Figure 4b). The cortex carries most of the compressive load, but the foam is able to accommodate and release deformed cortical flexure [83]. The presence of the foam increases the critical flexural strength, flexural strain, and elastic strain energy absorption. The foam diameter decreases from the center to the cortex, and the foam deformation plays an essential role in adapting to the local flexion of the cortex. The foam exhibits extensive tensile and compressive deformation around the flexural cortex [1]. The reinforcement tendons within the quills extend from the cortex to the center. In synthetic sandwich structures, the foam is usually easily separated from the outer cortical layer. However, the strong connection between the foam and the cortex endows the porcupine quills with superior tear resistance compared to synthetic sandwich construction. The foam structure of porcupine quills can provide inspiring clues for the design and manufacture of biomimetic composites with lightweight buckling-resistant properties [3,11,83].

Birds need to reduce their body mass to ensure flexibility and stability in flight [5]. Thin-walled cylindrical structures have evolved in several parts of the bird’s body [1], such as the beak (Figure 4c). Beaks generally are divided into two categories: short and thick ones or long and thin ones [1]. However, the Toco Toucan (*Ranifastoridae*) is a notable exception. Its beak is a third of its length and requires a fairly thick bill for foraging and fencing activities in the canopy. The bill accounts for only one-thirtieth of the bird’s total weight and has an extremely low density of 0.1 g/cm^3^ [86]. The beak is quite a delicate organ with an external cuticle and an internal osteocyte structure. The cells consist of osteoclasts and are connected by membranes [86]. The foam with a hollow inside is another distinctive feature of the Toucan beak, leading to further weight reduction [1,84].

The pterygoid cross-section of the wandering albatross characterized by micro-computed tomography (micro-CT) is clearly different at varied locations (Figure 4d) [15]. The diversity of bone segments ensures that various locations with different mechanical properties can be subjected to different loads. By precisely controlling the cross-section shape, the bird’s wing bone achieves local properties that closely match the specific local working conditions. Meanwhile, the bone has an internal hollow lattice structure and ridge structure. Ridges are protrusions on the inner wall of the bone and typically form at −45° to the horizontal axis of the bone, which exist in danger regions with local buckling due to the high combined bending and torsional loads. The bone with these structures can resist the large tensile stresses along the directions when torsion occurs [15].

The flight feathers of birds have been proven to be an extreme design consideration for the stiffness-to-weight ratio [15]. A single feather consists of a shaft and barbs distributed with delicate secondary structures: barbules. The shaft can be further divided into a cortex and a medulla filled with closed-cell foam. The inner walls of the medulla are also made of foam in the second level of porosity, which further reduces the feather density [13,87]. The whole feather consists of keratin, and the cortex can be regarded as fiber-reinforced composites with multilayer structures [15,88,89]: (1) crystalline *β*-keratin filaments are embedded in amorphous matrix proteins at the sub-nanostructure (~3 nm in diameter); (2) filaments bundle are encased in amorphous inter macrofibrillar (50–400 nm in diameter); (3) macrofibrils assemble to form fibers (3–5 μm in diameter); (4) fibers establish ordered lamellae within the feather shaft cortex (hundreds of microns). Lingham-Soliar et al. [90] found ordered fibers and macrofibrils in the shaft. Later studies showed that the arrangement of fibrous keratin composites differed between species, possibly based on the flight style of birds. Wang et al. [89] found that the outer layer of California gull (*Larus californinicus*) feathers had a thin encircling layer of fibers with a thick inner layer of longitudinal fibers on the dorsal side of the calamus and proximal shaft of the feather. This fiber arrangement is commonly used in composite designs where axial fiber separation is suppressed by preventing axial splitting during flexure. Crossed fibers with an angle of ±45° on the side of the feather shaft are subjected to major shear stresses. In torsion, the fibers are aligned at 45° of the shafts along the axial stresses, and they increase the torsional stiffness. Inspired by the microstructures of feathers, bioinspired composites with lightweight and high-stiffness properties can be designed and fabricated (Figure 4e) [15,89].

## 5. Design and Fabrication of Bioinspired Composites

Due to the excellent mechanical properties of biomaterials, the design and fabrication of bioinspired composites have attracted a great deal of attention [4,7,11]. The bioinspired composites are achieved by imitating the structure, interface, and synthesis process of biomaterials [11,12,95,96,97]. It is not by mindlessly copying nature but by relying on the intrinsic advantages of artificial materials while drawing on the excellent characteristics of organisms to achieve the purpose of “from nature, beyond nature” [5]. In recent years, bionic technology has been more widely used in the optimization design of fiber-reinforced composites due to its ingenious ideas and innovative concepts. In the fabrication process of bioinspired composites, the structure and interfacial properties of biomaterials are commonly imitated [5].

**Figure 5:**
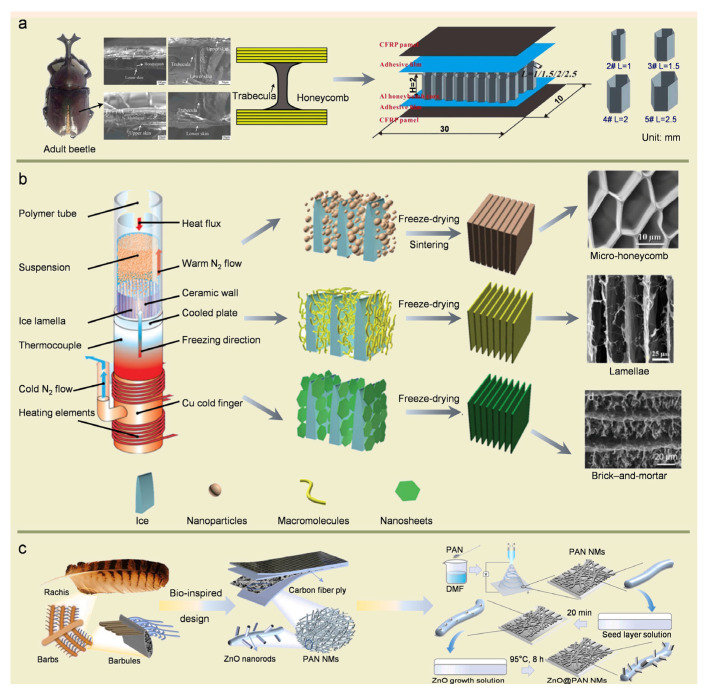
(**a**) Engineering mapping and rational design of bionic structural material (BSM) inspired by the beetle forewing. Reproduced with permission from Z.-B. Cai et al. [98], Elsevier. (**b**) Preparation of bionic cellular structures using the freeze-casting technique. Reproduced with permission from M.-A. Shahbazi et al. [101], John Wiley and Sons. (**c**) Design strategy and fabrication method of bioinspired carbon fiber reinforced polymer (CFRP) composites. Reproduced with permission from W.D. Song et al. [109], Elsevier.

In terms of structures, Cai et al. [98] designed and fabricated structural composites with good impact resistance inspired by the beetle sandwich structure, the energy absorption rate of which increased by 175% compared with conventional fiber-reinforced composites (Figure 5a). He et al. [99] incorporated short-cut fibers trabeculae or long fibers trabeculae structures in honeycomb panels inspired by the beetle structure, which exhibited good mechanical performance. Inspired by the woodpecker skull, Abo Sabah et al. [100] designed and fabricated a bioinspired sandwich beam with carbon fiber reinforced composites as the skin, aluminum honeycomb as the core, and rubber as the interface materials between the skin and the core. The damaged area of the bioinspired sandwich beam was reduced by 60–95% compared with the traditional beam after adding the rubber structure. The impact resistance index is 2.7–5.7 times higher than that of traditional beams, which have excellent low-speed impact resistance.

Freeze-casting and additive manufacturing is widely used as emerging fabrication methods to obtain bioinspired structures [20]. The freeze-casting method mainly uses growth ice as a template to build various structures by controlling the freezing conditions to form layered microstructures in the space between ice crystals. In addition, various factors can be controlled to modulate the local structural features and further control the local properties by changing the slurry concentration, freezing rate, sintering, temperature gradient, etc. For example, the unique pearl layer structure can be replicated by intelligently adjusting the temperature gradient [5,20,101] (Figure 5b).

Due to the high degree of freedom in structure design, the 3D printing technique is becoming a powerful additive manufacturing technology to produce 3D structures with arbitrary geometries at micro-/macroscales [7,11,21,102,103,104,105]. Mao et al. [40] investigated the complex structure of cuttlebone and revealed its role in obtaining high porosity and excellent mechanical properties. Based on this, a cuttlebone-inspired lattice material was developed by 3D printing. It was characterized by lightweight, high strength, and high energy absorption capacity, which has promising applications in many fields, such as aerospace structures and implantable devices.

The interfacial properties in the reinforcing phases and matrix of biomaterials can effectively be adjusted and contribute significantly to their mechanical performance [5,20]. Therefore, imitating the interfacial properties of biomaterials is an additional effective approach to improve the mechanical performance of bioinspired composites. Inspired by the dactyl club of mantis shrimp, the layered design of fibers enhances the impact resistance of fiber-reinforced composites [106,107]. Inspired by the flight feathers of birds, the modification of fiber surface or interlayer design improves the strength and toughness of fiber-reinforced composites [108,109] (Figure 5c). Inspired by the byssus cuticle of marine mussels, a microphase-separated structure was introduced to effectively enhance the fracture toughness of the resin [110,111].

## 6. Summary and Outlook

In this review, based on recent substantial research advances on excellent biomaterials, typical lightweight structural biomaterials are selected and categorized into three groups based on their basic structure forms: cellular structure materials, fibrous structure materials, and sandwich structure materials. These structures and their mechanical properties, as well as potential applications, are discussed. Inspired by these novel structures, the design strategy and fabrication method of advanced materials with lightweight and high mechanical performance are also summarized briefly. Herein, several points need to be highlighted to provide clues for future research on biological prototypes and high-performance bioinspired composites.

(1) There is still significant investigation potential on the interior synergistic processes of lightweight and high mechanical performances of biomaterials. Elaborate theoretical models need to be established to reveal the complicated relationship between structures and mechanical performance of biomaterials. Moreover, developing the on-demand trade-off strategy between lightweight properties and high mechanical performance is the eternal theme for advanced bioinspired composites.
(2) More professional techniques are required to expose the microscopic characterization and inherent biological mechanisms of biomaterials, such as micro-CT, digital image correlation (DIC), and in situ testing. Moreover, due to the inherent complexity of biomaterials, it is necessary to consider various influencing factors when researchers conduct a deep investigation on biomaterials. To precisely predict the behaviors of biomaterials under external force, accurate numerical simulation is a powerful tool for researchers to understand the inner change mechanism of biomaterials. It could provide a more comprehensive and profound elaboration for the uncovered mechanism and discover specific characteristic parameters for future bionic design.
(3) Inspired by excellent biomaterials, how to achieve the rational design and precise fabrication of bioinspired composites is another sustainable topic. To achieve twice the result with half the effort in bionic design, it is necessary and effective to investigate the integration mechanism of lightweight and high mechanical properties within living things. Reliability and simplicity should be maintained during fabrication to minimize the influence of the manufacturing process on the bionic design.
(4) A multidisciplinary collaboration involving bionic science, materials engineering, and chemistry opens up new avenues for the deep investigation of biomaterials, possibly breaking the bottleneck in the developing techniques of advanced bioinspired composites.

## Data Availability

Data sharing is not applicable to this article as no new data were created or analyzed in this study.
